# Where are the NGOs and why? The distribution of health and development NGOs in Bolivia

**DOI:** 10.1186/1744-8603-8-38

**Published:** 2012-11-23

**Authors:** Lindsay P Galway, Kitty K Corbett, Leilei Zeng

**Affiliations:** 1 Faculty of Health Sciences, Simon Fraser University, Blusson Hall 8888 University Drive, Burnaby, BC, V5A 1S6, Canada; 2 Department of Statistics and Actuarial Science, Waterloo University, 200 University Avenue West, Waterloo, ON, Canada

**Keywords:** Nongovernmental organizations, NGOs, Global health and development, Bolivia, Multiple regression, Count data

## Abstract

**Background:**

The presence and influence of nongovernmental organizations (NGOs) in the landscape of global health and development have dramatically increased over the past several decades. The distribution of NGO activity and the ways in which contextual factors influence the distribution of NGO activity across geographies merit study. This paper explores the distribution of NGO activity, using Bolivia as a case study, and identifies local factors that are related to the distribution of NGO activity across municipalities in Bolivia.

**Methods:**

The research question is addressed using a geographic information system (GIS) and multiple regression analyses of count data. We used count data of the total number of NGO projects across Bolivian municipalities to measure NGO activity both in general and in the health sector specifically and national census data for explanatory variables of interest.

**Results:**

This study provides one of the first empirical analyses exploring factors related to the distribution of NGO activity at the national scale. Our analyses show that NGO activity in Bolivia, both in general and health-sector specific, is distributed unevenly across the country. Results indicate that NGO activity is related to population size, extent of urbanization, size of the indigenous population, and health system coverage. Results for NGO activity in general and health-sector specific NGO activity were similar.

**Conclusions:**

The uneven distribution of NGO activity may suggest a lack of co-ordination among NGOs working in Bolivia as well as a lack of co-ordination among NGO funders. Co-ordination of NGO activity is most needed in regions characterized by high NGO activity in order to avoid duplication of services and programmes and inefficient use of limited resources. Our findings also indicate that neither general nor health specific NGO activity is related to population need, when defined as population health status or education level or poverty levels. Considering these results we discuss broader implications for global health and development and make several recommendations relevant for development and health practice and research.

## Introduction

Nongovernmental organizations (NGOs) have become increasingly important players in the realm of global health and development. They operate projects in low and middle-income countries (LMICs) throughout the world. Their number has grown twenty-fold since 1951 and increasingly donors prefer to channel aid to and through NGOS rather than directly to foreign governments [1]. In 2006, transfers of official development aid to NGOs totaled more than $2 billion (US) according to OECD statistic and services such as education and health care, historically managed and implemented by the public sector, are increasingly provided by NGOs [2,3].

Despite the growing prominence of NGOs in LMICs, the ways in which these organizations influence and are influenced by the context in which they work is not well understood. NGO research has emphasized exploration of the impacts of individual organizations or projects, often overlooking the broader implications of NGOs and their work [4,5]. Relative to their importance, systematic and empirical analyses of NGOs in global settings are few [6]. Research on the distribution of NGO activity, factors that are related to their distribution, and critical discussions regarding the implications of their distribution are limited [5]. Exploring NGO geographies and related factors highlights inequalities in resource distribution and can help us understand why NGO related resources flow to certain places and not others. To ensure that NGOs have positive impacts and that resources are allocated and used in ways that create positive and lasting change on the ground, more comprehensive, in depth, and critical explorations of NGOs and the NGO sector are needed.

This paper explores the distribution of NGO activity and related factors from a global health and development perspective. We address this issue through exploration of NGO activity in Bolivia. The overriding question is: what factors are related to the distribution of NGO activity across municipalities in Bolivia?

## Background

### The evolution of NGOs in global health and development

The growth of NGOs has been reviewed by various scholars [7-10]. The dramatic rise of NGOs is related to an increase in funding to and through NGOs, which reflects the largely untested assumption that NGOs are more cost-effective and better than the public sector in reaching poor and vulnerable populations [10-12].This assumption is rooted in the neoliberal ideologies dominant during the 1980s that promoted privatization, marketization, and a reduced government presence [13,14]. During this era, structural adjustment programs were imposed on indebted countries by international financial institutions in hopes of stabilizing economies and infusing neoliberal agendas into LMICs [13]. Although structural adjustment requirements varied across nations, a common theme was the dramatic reduction of government spending on social programs such as housing, health care, and education [13]. With efforts to decrease the role of the state and growing confidence in the free market, NGOs were touted as the most appropriate means to fill gaps in public services and hailed as development alternatives [15]. Greater space for NGO projects and increased confidence in the NGO sector relative to LMICs governments initiated the dramatic rise of NGOs throughout the world.

The evolution of the NGO sector can be characterized as four successive waves [7,8]. The characteristics of each of these four waves are summarized in Table 1.

**Table 1:** Summary of four waves of NGO sector evolution

**Wave**	**Approximate era**	**Defining characteristics**
**1st**	WWII to late 1960s	• Small number of large, well established NGOs
		• Primarily involved in emergency and conflict relief
		• Religious and missionary affiliations commonplace
**2nd**	Early 1970s to early 1980s	• Role expanded beyond emergency and conflict relief
		• Community level work employing participatory approaches commonplace
**3rd**	Mid/early 1980s to late 1990s	• Rapid proliferation of NGOs around the globe linked to changing political and economic ideologies of the time
		• Significant increase in funding for NGO related projects
		• NGOs were considered development alternatives and become the ‘favoured child’ of development agencies
**4th**	Early 2000s- current	• NGO activity shaped by poverty reductions agendas and Millennium Development Goals
		• Funders increasingly channelling aid to and through NGOs
		• Increase in bilateral aid and global health initiatives (i.e.Global Fund, PEPFAR)

### Global and national distribution of NGO’s

Over the last three decades, NGO activity has become unevenly distributed across continents and countries [2,5,6,16-19]. Currently, there is a paucity of research exploring the unevenness of NGO activity within nations. Bebbington (2004) remarks “there have been few serious attempts to map unevenness [of NGO activity] at this scale”. This is largely due to the lack of national NGO data. Few nations keep a representative registry of NGOs, and national surveys of NGO work have been limited and are costly. Studies that have examined the spatial distribution of NGOs at the national level have documented hotspots and blind spots of NGO activity [6,20-22]. In Tanzania for example, a nationally representative survey showed that NGOs are highly concentrated in the Arusha region while limited in others [21]. There are no published studies examining NGO distribution and related factors across regions in Bolivia, which is the focus of the case study described below.

### Factors related to NGO distribution

Description of NGO distribution, whether at the global, national or local level, can highlight regions where NGO activity is concentrated compared to regions where NGO activity is limited or non-existent. It is important to take this spatial analysis a step further and identify factors that are related to patterns of NGO activity. A literature search identified factors commonly cited as correlates of NGO activity geographies.

Poverty is the most important and commonly cited factor thought to be related to levels of NGO activity [6,20-23]. Since the poverty reduction agenda has become dominant within the NGO sector in recent years, we would expect to see more concentrated NGO activity in poor nations/regions and less in nations/regions that are more affluent. The few studies addressing this issue empirically have come to opposing results with respect to the relationship between poverty levels and NGO activity, such that the nature of this relationship remains unclear. Furthermore, the relationship between poverty and NGO activity has not been sufficiently tested at the national level.

A second commonly cited factor related to NGO distribution is the health and development needs of a population. Social and economic indicators of these needs include literacy rates, infant mortality rates, or life expectancy. Organizations whose objectives are oriented towards improving the well-being of neglected populations logically would target populations with serious needs according to health and well-being indicators. In a similar vein, NGOs commonly focus their efforts on sub-sectors of a population that are considered most vulnerable [23]. This suggests that NGO distribution would be in part determined by the distribution of vulnerable populations. Across different settings, different populations might be considered vulnerable such as youth, the elderly, or certain ethnic groups. It is reasonable to expect that NGOs in Bolivia would focus their efforts in regions with large indigenous populations. Although commonly described, the relationship between NGO distribution and population need or population vulnerability has not been empirically tested.

Mercer (2002) suggests that the proliferation of NGOs has occurred to a greater extent in urban compared to rural spaces. According to this argument, organizations and their projects tend to concentrate in regions that are highly urbanized to ensure access to economic, infrastructural, and human resources needed to support the organization [22,24]. What could be called an urban bias has been documented in several countries including Ethiopia [25], Uganda [26], Vietnam [27] and Tanzania [21]. A related idea explored by Chambers (2008), is a potential ‘tarmac effect’ leading to spatial biases in health and development work, including NGO projects. He suggests that health and development work tends to be limited to areas that can be accessed by tarmac or paved roads; clearly, this idea is related to the idea of an urban bias [28].

Regarding the health sector, which is a primary focus of the NGO sector as a whole, it is often asserted that NGOs tend to work in regions with limited public health system coverage and service availability [10,29]. This argument is linked to the broader claim that is common within the NGO literature, and which has been an argument fuelling the proliferation of NGOs in general since the mid-1980s: that NGOs are well-situated to fill in the gaps left by the contraction of the public sector.

### The Bolivian context: Health, development, and NGOs in Bolivia

Bolivia, with a population of just over 10 million [30], is the poorest nation in South America. According to recent data, 23 per cent of the population live below the poverty line, the maternal mortality rate is 290 per 100,000, and it has one of the lowest indicators of human development in the western hemisphere (ranked 108th out of 169 UN member nations) [31,32]. There are widespread inequalities in resources and health outcomes. Indicators of health and well-being are substantially worse among poor populations. Looking at child mortality as an example, the absolute gap between the richest fifth of the population and the poorest fifth of the population is 82 child deaths per 1,000 births [33]. Addressing inequalities, reducing poverty, and improving health outcomes are primary concerns in Bolivia.

Bolivia attracts one of the highest volumes of aid per capita in South and Central America [34]. A large proportion of official development assistance is administered to and through NGOs that are working towards improving health, supporting development, and reducing poverty within the nation. The majority of NGOs working in Bolivia receive international funding; few NGOs operate with domestic support [35].

**Figure 1:**
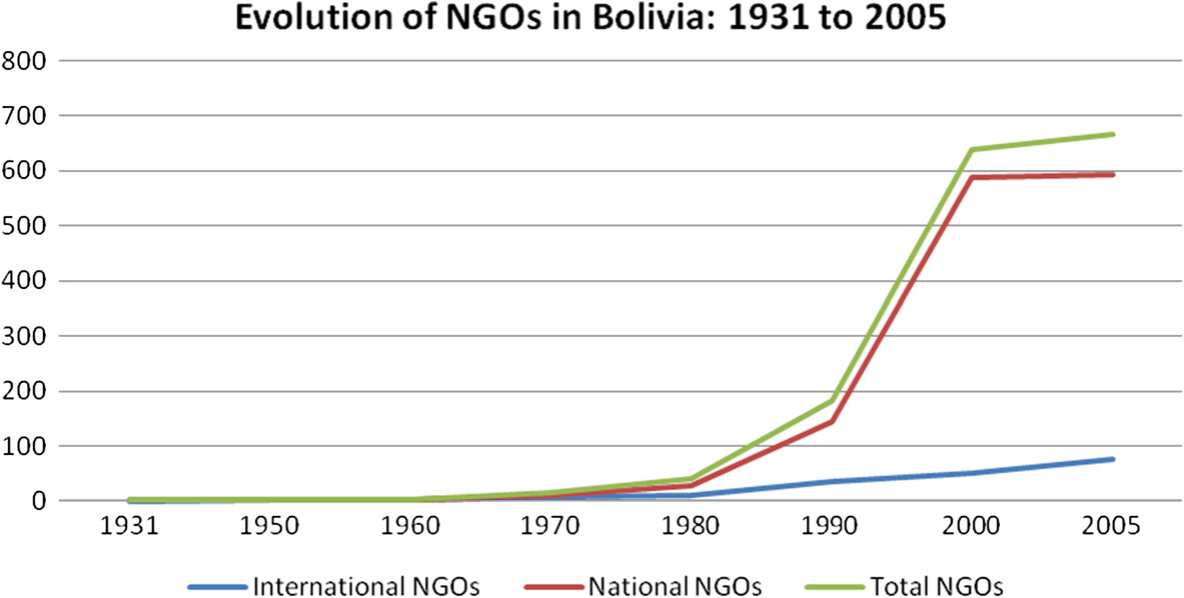
NGO evolution over time in Bolivia: 1931–2005.

The evolution of the NGO sector in Bolivia has followed the four waves of NGO evolution outlined in Table 1. NGOs were practically non-existent prior to the 1980s, after which the number of NGOs in Bolivia increased exponentially (see Figure 1). In 1980, government estimates suggest that there were only 39 NGOs working in the nation and upwards of 600 by the end of the 20th century [36]. The most dramatic expansion in scale, scope, and influence of NGOs in Bolivia occurred between 1985 and 1995.

The reasons underpinning the neoliberal paradigm that dominates the NGO and development sector in Bolivia are complex [37]. In 1986, Bolivia’s newly elected president implemented a Structural Adjustment Program entitled *La Nueva Politica Economica* (the New Economic Policy). This reform program was guided by neoliberal ideology and strongly (if not forcefully) encouraged by the international financial institutions [38]. Deregulation of product markets, liberalization of trade regimes, privatization of national industries, and a reduction of state spending in health and other service sectors were at the heart of the reform programme [39]. From the perspective of neoliberal economists, the international financial institutions, and international lenders, the Bolivian reform process was considered a success as the national economy stabilized and the role of the government had been reduced; Bolivia was coined a star reformer and ‘a model pupil’ of the International Monetary Foundation and The World Bank [38,40]. However, the government restructuring and economic reform process benefited relatively few while undermining the livelihood and well-being of the majority of Bolivians. Poverty levels increased and health and well-being of the population declined; while at the same time, NGOs were hailed as the most appropriated alternative to state programs for the economic and social needs of the population [9,41]. In this environment “NGOs were an attractive channel for international development assistance intended to reduce poverty and improve the well-being of the population” [41]. The increased space for NGOs in the absence of state programs and support, and the increased NGO funding from institutions like the World Bank and the WHO (as well as other donors), contributed to a dramatic increase in the intensity and coverage of NGOs and their projects in Bolivia. It is noteworthy that within the last decade, the proliferation of NGOs in Bolivia appears to have slowed (See Figure 1).

## Methods

We used data on NGOs in Bolivia first to affirm that NGO activity was unevenly distributed across the country, and then to assess the hypotheses that NGO activity across space is related to: (1) poverty levels, (2) levels of population need, (3) size of the indigenous population, (4) extent of urbanization, (5) population size, and (6) level of health system coverage.

### Data sources and variables

In this study, we define NGO in the same manner as the Bolivian government, since the question at hand is explored using its data. According to the Bolivian government an NGO is “any not-for-profit organization or group of people, both foreign and national, of religious character or not, that implements activities to improve well-being and development within the national territory, which may be financed by state funds or international co-operation resources” (translated from [36]). This definition is similar in scope and tone to the often cited definition offered by Vakil (1997), who writes that NGOs are best understood as “self-governing, private, not-for-profit organizations that are geared to improving the quality of life for disadvantaged people”.

The total number of NGO projects per municipality was selected as a proxy for NGO activity. The entire country of Bolivia is divided into municipalities; they are the third level of administration below departments and provinces. The term municipality therefore refers to “the smallest government unit in the country that holds regular elections, has the power to tax, and determines local policy and laws” [42]. The municipality was selected as the unit of analysis because this is the smallest administrative unit with decision-making authority and also the smallest unit for which economic, social and health indicators are consistent, adequately accurate, and recent, and therefore appropriate for statistical analysis [42].

Data regarding the total number of NGO projects per municipality were extracted from the 2005 National NGO Directory [36]. The Vice-Ministry of Public Investment and External Financing (VIPFE) has kept a registry of NGOs working in Bolivia since 1990 [36]. The published registry has been updated a total of five times; the 2005 National NGO Directory was the most recent. The government requires that all NGOs working in the country register as a legal entity; it is the responsibility of the NGOs themselves to register and information is recorded by the NGOs [43]. The level of detail and information collected has changed with each successive update of the registry. For this study, the 2005 National NGO Directory was downloaded as a PDF document from the VIPFE website and used to create a database consisting of the total number of NGO projects per municipality as well as the total number of NGO projects per municipality disaggregated by sector. The dataset also contains indicators for the explanatory variables of interest compiled from the 2001 national census. The explanatory variables are defined below.

1. *Poverty level*: Municipal poverty level is indicated by percentage of the population living in poverty according to the Unsatisfied Basic Needs (UBN) index. The UBN index is often used as an indicator for poverty level in Latin America. It is constructed by combining census level household measures including adequate housing conditions, access to water and sanitation, and availability of electricity into a composite indicator representing poverty for small administrative units [44]. A large body of literature exists on the strengths and weaknesses of the UBN as an indicator for levels of poverty (see [44,45]). We selected the UBN index over other possible indicators of poverty level because it is more comprehensive and data were available for all 314 municipalities.

2. *Population need:* We used two variables to operationalize population need: population health and education level. Population health is indicated by infant mortality rate (IMR) (total number of infant deaths per 1,000 live births) which is a commonly used indicator for population health. Percentage of the municipal population with a secondary school education is used to measure education level of the population.

3. *Size of the indigenous population.* In Bolivia*,* the indigenous population is considered highly vulnerable and is a marginalized population [46,47]. This variable is therefore also a proxy for vulnerability. The term indigenous refers to all indigenous groups in Bolivia considered together.

4. *Extent of urbanization*. The extent of municipal urbanization is measured by the percentage of the municipal population that lives in settlements designated as urban compared to rural. The Bolivian government defines its urban areas as settlements with more than 2,000 inhabitants [48].

5. *Population size:* This is indicated by the total population of the municipality. This explanatory variable is log transformed in the model due to large variance across units of analysis.

6. *Health system coverage.* The percentage of women receiving antenatal care at least once during pregnancy is often used as an indicator of health system coverage within the field of public health [49]. This indicator is also related to access to services but can be used as a proxy of public system coverage.

Data for these variables were taken from the 2001 national census (from the National Institute of Statistics -El Instituto Nacional de Estadística). Indicators for the explanatory variables of interest were selected for the year 2001 (about four years prior to the NGO activity data) rather than the most recently available (2006). We did this for two reasons. First, this ensured that analyses examined how NGO distribution is influenced by the characteristics of the municipality and not the other way around. If data for both the dependent and explanatory variables were collected from the same year, it is possible that the identified statistical relationships were due to the influence of NGO activity on the explanatory variables rather than the influence of the explanatory variables on NGO activity. If, for example, a significant relationship was found between poverty levels and NGO activity using 2005 data for both variables, it would not be possible to say whether the poverty levels influenced NGO activity or vice versa. A four-year lag between the explanatory and dependent variables may address this issue. As well, the 2001 data are rich and complete compared to more recent years such that there were no missing data.

### Analyses

Preliminary analyses included visual analysis of the spatial distribution of NGO activity across municipalities using a geographic information system (GIS) (version 9.3.1 Environmental Systems Research Institute, Inc. Redlands, California, 2009) and descriptive analyses including univariate and bivariate analyses on response and explanatory variables. The GIS was used to create maps that visually represent the spatial distribution of NGO activity as well as the distribution of population size, poverty levels, population need, extent of urbanization, size of vulnerable population, and health system coverage. Maps displaying both municipal NGO activity and each of the explanatory variables were created to visually examine crude relationships. A bivariate analysis employing Spearman’s rank coefficient was used to examine pair-wise associations between the explanatory variables of interest and NGO activity per municipality. This indicated which explanatory variables were correlated with NGO activity without marginally controlling for the effects of other variables, and it was also used to assess multicollinearity among explanatory variables. Multi-collinearity occurs when two or more independent variables are highly correlated with each other which can lead to unreliable estimations for the standard errors of the regression coefficients and therefore confusing and misleading results. Multi-collinearity was not detected since no variables were strongly correlated to each other (no maximum or minimum correlation coefficients greater than 0.80 or less than - 0.80).

A multiple regression analysis of count data was conducted to examine the relationship between NGO activity and the multiple explanatory variables of interest. Regression models were built for 1) the total number of NGO projects and 2) a subset of health sector NGO projects. The purpose of the latter, health sector specific model was to examine whether similar associations existed within this subset of health specific NGO projects and to test the hypothesis that coverage of the public sector, specifically the public health system, is related to NGO activity.

Since NGO activity is indicated by count data, a multiple Poisson log-linear regression model was selected as a starting place for the model building process. The Poisson distribution is commonly used to model count and rate data [50]. If let N_*i*_ indicate the number of NGO activities in the *i*th municipality and λ_*i*_ indicate the mean number of NGO activities in the *i*th municipality, we assume that N_*i*_ follows a Poisson distribution with the following density:

$$P\left(Ni=ni\right)=\frac{{}^{{-}_{\lambda }^{i}}\lambda_{i}^{\mathrm{ni}}}{\eta_{i}!}\;,\eta_{i}=1,2$$

Then the explanatory variables are linked to the mean count λ_*i*_ through a log-linear regression model as follows:

Model 1: All NGO Activity

log (λ_*i*_) = β_0_+β(Poverty level _*i*_)+β_1_( Population health _*i*_)+β_2_(Education level _*i*_) _*+*_ β_3_(Extent of urbanization _*i*_ )+β_4_(Size of indigenous population _*i*_) +β_5_(Population size _*i*_ )

Model 2: Health NGO Activity

log (λ_*i*_) = β_0_+β(Poverty level _*i*_)+β_1_( Population health _*i*_) +β_2_(Education level _*i*_) *+* β_3_(Extent of urbanization _*i*_ ) + β_4_(Size of indigenous population _*i*_) + β_5_(Population size _*i*_ ) + β_6_(Health system coverage _*i*_ )

The regression coefficient β, represents the expected change in the log of the mean count of NGO projects associated with per unit change in the explanatory variable.

A limitation of the Poisson distribution is that the variance of the data is restrained to be equal to the mean [50]. When this is not true, the data are characterized as being either over-dispersed or under-dispersed. Over-dispersion is a common problem and leads to inaccurate estimations of regression coefficient standard errors that can influence the precision of the hypothesis test results. The data in both model 1 and 2 were found to be over-dispersed as indicated by deviance factors greater than 1. To address the identified over-dispersion, a negative binomial distribution was assumed for the NGO activity count instead. The negative binomial distribution can account for the over-dispersion of count data as it is not restricted to having the variance equal to the mean [50]. Additionally, a formal test, the likelihood ratio test for significance of over-dispersion, was conducted which also supported the use of the negative binomial model. The interpretation of the regression coefficients when using the negative binomial model is the same as that for the Poisson model as described above.

Goodness-of-fit of the final models were evaluated visually by comparing the estimated cumulative probability distributions from the negative binomial model to the observed cumulative probability distribution and comparing the value of the deviance factor statistic and the Pearson’s chi-squared statistic to 1. The model was also checked for outliers using plots of Pearson’s residuals and the deviance residuals (which measure the relative deviation between the observed count data and estimated count data) against observation number.

For the purpose of this study, we considered a p-value of less than 0.05 as highly significant and a p-value less than 0.10 as weakly significant.

All statistical analyses were performed using the statistical software package SAS (version 9.2, SAS, Institute INC. NC, 2006).

## Results

**Figure 2:**
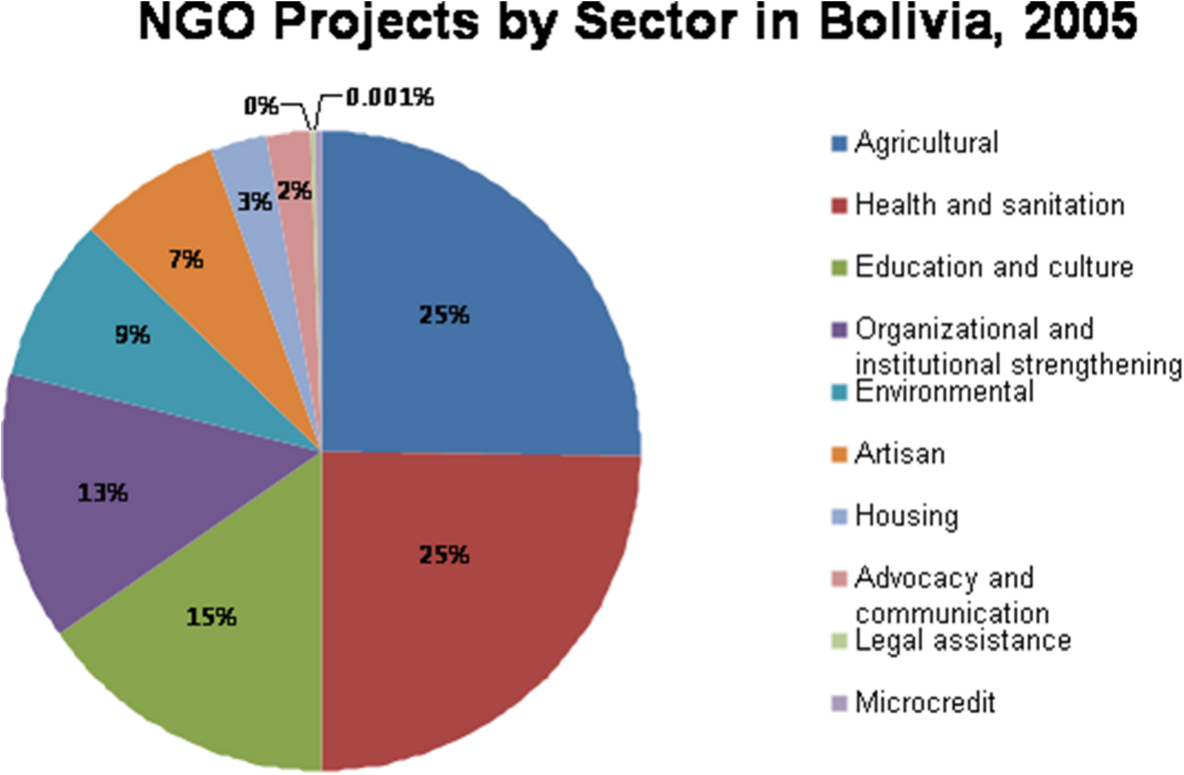
NGO projects by sector in Bolivia: 2005.

In 2005, the 667 NGOs working in Bolivia were conducting a total of 4,482 projects. Figure 2 shows the proportion of NGO projects working in each of the eleven NGO sectors (sectors classified by the Bolivian NGO Directory). The greatest proportion of NGO projects were classified in the health and agriculture sectors; each comprising twenty-four per cent of the total NGO projects and together accounting for nearly half of all NGO projects. Microcredit, legal assistance, advocacy and communication, and housing projects together account for less than eight per cent of all NGO projects.

Characteristics of the 314 municipalities are summarized in Table 2. The average number of NGO projects across all Bolivian municipalities is fourteen projects per municipality. Twenty-seven municipalities (8%) have no NGO projects while the total count of NGO projects is greatest for La Paz (the capital municipality) with a total count of 313. The mean number of health and sanitation NGO projects per municipality is three projects.

**Table 2:** Descriptive statistics for explanatory variables, N = 314

**Explanatory variable**	**Indicator**	**Mean**	**SD**	**Minimum**	**Maximum**
**NGO activity**	**Total # of NGO projects, 2005**	14.27	28.82	0.00	313.00
**Health sector NGO activity**	**Total # of NGO projects in the health and sanitation sector, 2005**	3.44	6.72	0.00	74.00
**Poverty level**	**Percentage of municipal population with unsatisfied basic needs, 2001**	84.23	17.88	19.08	100.00
**Population need a)Population health status**	**Infant mortality rate, 2001**	76.50	22.38	20.00	170.00
**b)Education level**	**Percentage of municipal population with secondary school education, 2001**	67.71	15.24	10.16	98.79
**Size of the indigenous population**	**Percentage of municipal population that is indigenous, 2001**	45.00	31.00	0.00	87.00
**Extent of urbanization**	**Percentage of municipal population that is urban, 2001**	19.00	28.00	0.00	100.00
**Population size**	**Natural log of municipal population, 2001**	9.17	1.24	5.40	13.94
**Health system coverage**	**Percentage of women receiving antenatal coverage, 2001**	40.40	23.51	0.00	100.00

*SD* = standard deviation.

**Figure 3:**
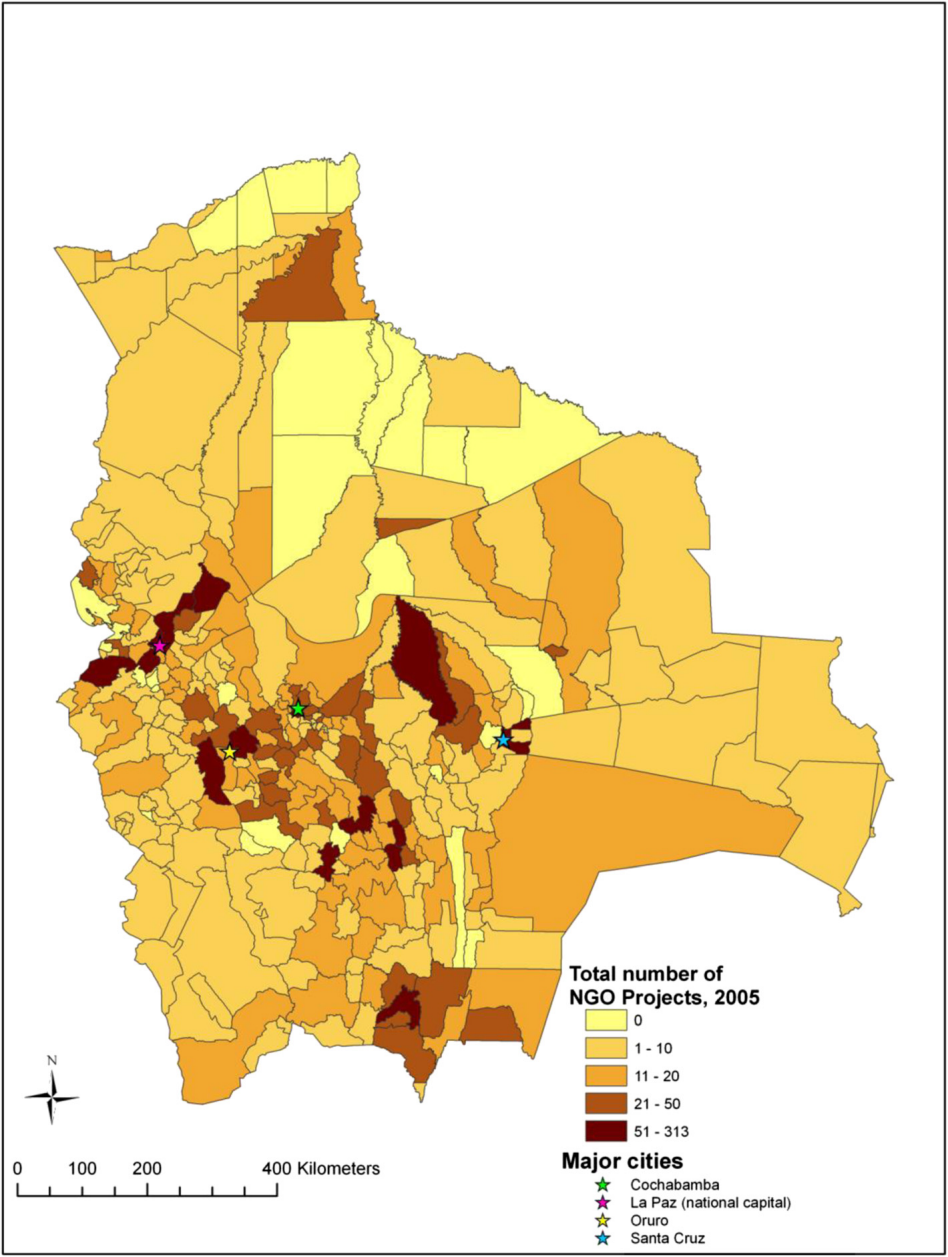
Map of NGO activity across Bolivian municipalities.

**Figure 4:**
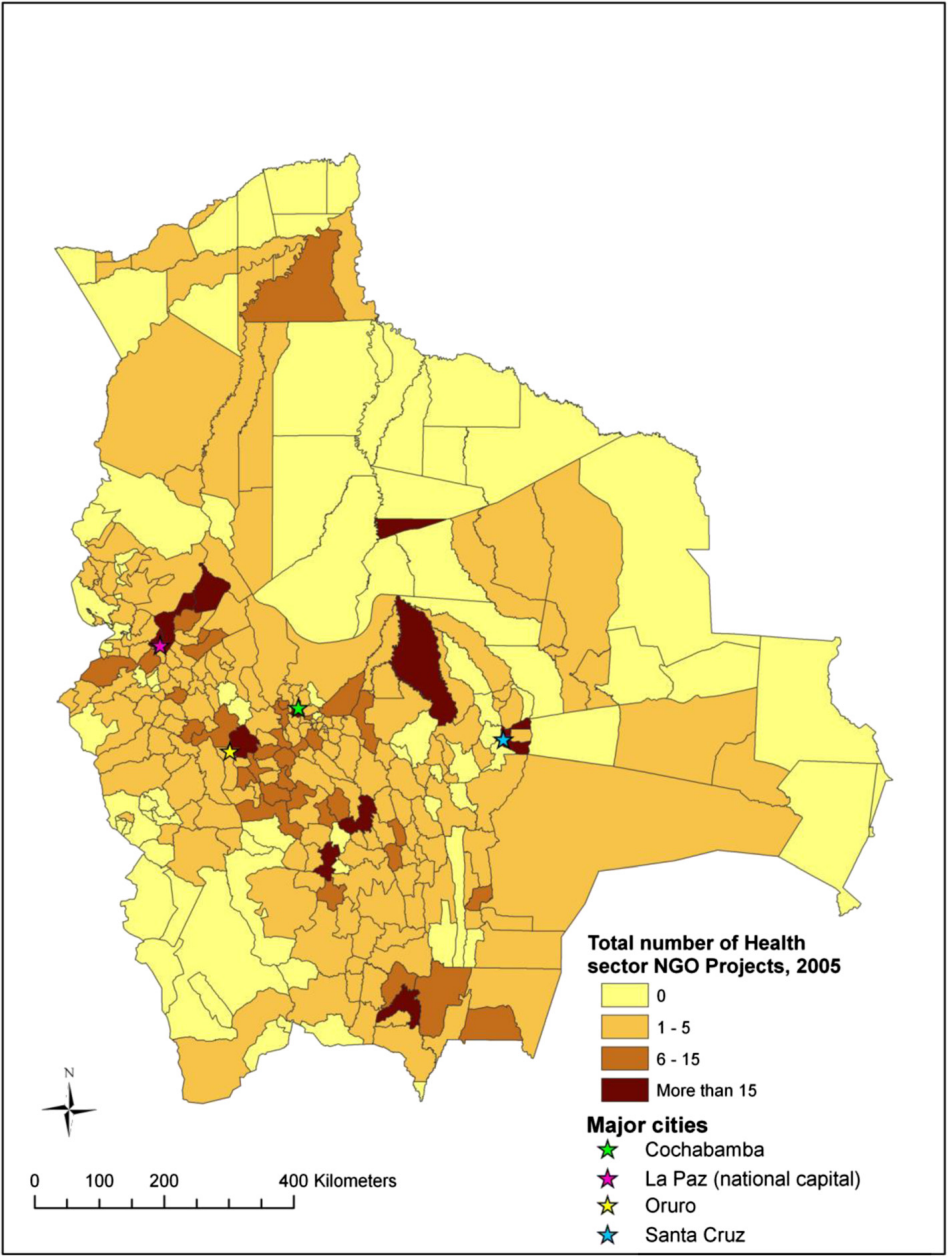
Map of health sector NGO activity across Bolivian municipalities.

Figure 3 illustrates the distribution of NGO activity across Bolivian municipalities and Figure 4 represents the distribution of health sector NGO activity across Bolivian municipalities. These figures highlight those municipalities where NGO activity is concentrated compared to those where NGO activity is limited.

The multiple regression analyses identified those explanatory variables that are related to NGO activity and health sector NGO activity across municipalities while adjusting for the effects of all other variables in the model. Table 3 summarizes the results of the final negative binomial log-linear regression for model 1, all NGO activity, and model 2, health sector NGO activity. Model 1 shows that when controlling for the effects of all other variables in the model, population size, extent of urbanization, and size of the indigenous population are significantly related to municipal NGO activity while all other variables are not. Both population size and size of the indigenous population are highly and positively related to municipal NGO activity (0.7554; p-value = <0.0001 and 0.7214; p-value = 0.0003) while extent of urbanization is negatively related to municipal NGO activity (−0.0054; p-value =0.067).

**Table 3:** Summary of regression models

	**Model 1: All NGO activity**			**Model 2: Health Sector NGO activity**		
**Variable**	**b**	**SEb**	**Wald p-value**	**b**	**SEb**	**Wald p-value**
**Poverty level**	−0.0058	0.0045	0.1970	−0.0072	0.0052	0.1703
**Population need**	0.0004	0.0026	0.8937	−0.0018	0.0034	0.5842
**a)Population health status**						
**b) Education level**	−0.0016	0.0036	0.6639	−0.0013	0.0046	0.7721
**Extent of urbanization**	−0.0054	0.0030	0.0673*	−0.0060	−0.0036	0.0890*
**Size of the indigenous Population**	0.7214	0.0020	0.0003**	0.0081	0.0026	0.0026**
**Health system coverage**	n/a	n/a	n/a	0.8166	0.0025	0.0010**
**Population size**	0.7554	0.0536	<0.0001**	0.7058	0.0665	<0.0001**

**significant at 0.05;

* significant at 0.10;

**b** = beta coefficient;

SEb = Standard Error of beta coefficient.

In model 2, population size (0.7058; p-value = 0.001), extent of urbanization (−0.006; p-value = 0.08), and size of the indigenous population (0.8116; p-value = 0.0026) remained significantly related to health sector NGO activity. Additionally, health system coverage was positively and significantly related to health sector NGO activity (0.0082; P-value = 0.001).

The criteria for assessing goodness of fit (deviance and Pearson χ ^2^ statistics) indicate that that the models fit the data well (deviance and Pearson χ ^2^ statistics close to 1).

### Limitations

The most important limitation is related to the definition, operationalization, and interpretation of the dependent variable ‘NGO activity’ as indicated by the total number of NGO projects in a municipality. First of all, qualitative and quantitative characteristics vary across NGOs and NGO projects but, aside from sector and location, heterogeneity in NGO characteristics is not captured in the data. Further, each NGO project is operationalized and counted equally (all weighted as 1 in other words), which ignores real differences in size, reach, scope, mission, and budget related to the project. As an example, consider the construct of project reach, an element within the RE-AIM evaluation framework defined as “The absolute number, proportion, and representativeness of individuals who are willing to participate in a given initiative, intervention, or program” [51]. Some projects may involve small numbers of people while others engage with many, thus having much larger reach. Due to the nature of the data available, it is not possible to comment on reach. These are important limitations that are however, unavoidable given the level of detail and scope of NGO related data that is available. Nonetheless, these data are a unique and valuable resource for exploring questions related to NGO activity.

Additionally, although efforts are made to register all NGOs working in the nation, accuracy of the data is unknown. A primary goal of the National NGO Directory, as stated by the Bolivian government, is to provide information that is reliable and accurate [36]. Since it is the responsibility of the NGOs themselves to register and the state does not actively monitor registration, it is likely that some NGOs have not registered. If NGO information is missing randomly rather than systematically, it would not influence the analysis towards significant but inaccurate results.

The explanatory variables were extracted from national census data. Census data are always subject to potential bias related to sampling methods.

Lastly, we have classified municipalities as rural versus urban according to Bolivian standards where a settlement of 2,000 or more inhabitants is classified as urban. We acknowledge however, that an urban setting with 5,000 inhabitants is qualitatively different from a large urban setting with a population of several hundred thousand people. The classification of regions into urban and rural status is a common challenge in empirical studies.

In spite of these data limitations, this paper remains a valuable contribution to the literature and advances our understanding of the determinants of NGO activity across space in Bolivia.

## Discussion

This study provides one of the first empirical analyses exploring factors related to the distribution of NGO activity at the national scale. Our analyses show that NGO activity in Bolivia, both in general and health sector specific activity, is distributed unevenly across the country. Similar to what has been shown at the global level [18] as well as at the national level for a handful of other LMICs [6,20-22], hotspots and blind spots can be identified on the maps of NGO activity in Bolivia. NGO activity tends to be most concentrated in those municipalities located in the central highland region while quite limited in the north-eastern lowland region. This uneven distribution may suggest a lack of co-ordination among NGOs working in Bolivia. Although a handful of NGO networks do currently exist (i.e. PROCOSI, a network of 31 NGOs that provide health services in the country), generally speaking, co-ordination of NGOs and their projects ought to be improved. In the experience of the lead author of this paper, NGOs working in Bolivia can be unaware of similar projects and can even duplicate of programs within a small geographic region. Co-ordination of NGO activity is most needed in regions characterized by high levels of NGO activity in order to avoid duplication of services and programmes and inefficient use of limited resources. Considering recent advances in communication technology, social media, and online mapping, great opportunities exist for improving co-ordination among those NGOs working in a given nation or region.

It is also likely that the uneven geographies of NGO work is a function of poor harmonization among donors that fund NGO work. In 2005, The Paris Declaration on Aid Effectiveness emphasised harmonization among donors as a key principle for greater aid effectiveness in LMICs [52]. Bolivia was among the 100 signatories of The Paris Declaration on Aid Effectiveness in 2005 and also one of ten countries that participated in a national-level evaluation of the declaration in 2007. This study documented generally poor results with regards to harmonization in Bolivia and suggested that greater efforts are needed at the level of the funders to improve well-being and quality of life on the ground [53]. More research is needed to better understand the ‘architecture’ [54] of NGO funding and to identify means to improve the organizational, management, and decision-making structures that co-ordinate funding.

Our analyses suggest that NGO activity is related to municipal population size: there is greater NGO activity in the more populated municipalities. This is true for both NGO activity in general and health sector NGO activity. Interestingly, although the literature highlights an urban bias in NGO activity in other settings [21,25-27], the opposite was found in this study. General and health specific NGO activity is higher in those municipalities that are more rural compared to those municipalities that are more urbanized. The divergence between our findings and the urban bias documented in other settings is likely explained by the use of NGO projects as an indicator for NGO activity in our study rather than total number of organizations used in most other studies. NGO head offices often exist in urban center; in Bolivia they are commonly located in La Paz, Cochabamba, and Santa Cruz while the projects they implement tend to take place outside of these urban centers in settlements characterized as rural. This highlights the importance of using NGO projects rather than organizations to measure and monitor NGO activity.

This study did not find a relationship between municipal poverty levels and NGO activity in Bolivia, suggesting that NGOs do not target the poorest regions in the country. This might indicate that NGOs are reluctant to work in the poorest regions. If that is the case, they might be responding to their sense that it is more difficult to demonstrate measurable success to funders when working in these environments which jeopardizes future funding and therefore organizational survival [6,21]. Many authors and practitioners have suggested that the ‘marketization’ of the NGO sector and recent focus on measurable impacts have had unfavourable side effects on the poorest regions by creating a bias towards those regions that offer ‘easier’ work environments [55,56]. Koch (2009) points out that, “the poverty orientation of NGOs and aid may be undermined by increasing pressure…to demonstrate project-related poverty impacts”. Bebbington (2004) suggests that evidence of this effect exists in the Andes where NGOs in Peru and Bolivia have expressed concerns about losing funding if they were unable to demonstrate short-term poverty impacts related to project implementation. This suggests that changes in funding structures and aid chains is called for to ensure that NGO related aid and projects reach the poorest populations [57]. Also, funders supporting NGOs must realize that funding conditions can have important implications for NGO decisions that may ultimately lead to undesirable consequences for the populations that both the donors and NGOS intend to help [58]. More research examining the importance of donor-NGO relationships and the impact of funding conditions on NGO work is called for.

Our findings also indicate that neither general nor health specific NGO activity is related to population need, when defined as population health status or education level. It may the case that factors other than need or perhaps perceptions of need determine where NGOs work [28,59].

This study thus does not find strong support for the widely held belief that the NGO sector targets the poorest and neediest regions or populations within a nation. Similar results have been found in other studies asking similar question in different settings [6,21,59]. These results are troubling considering that resources available for health and development efforts are limited and may not be reaching the most disadvantaged populations. It is possible however that NGO activity is related to relative levels of poverty or population need within a municipality rather than absolute municipal levels. For example, NGOs may focus their work on the poorest communities or individuals *within* a given municipality that is itself not characterized by high levels of absolute poverty. That said, Fruttero and Gauri (2005) examined the NGO sector in Bangladesh and found that community level poverty was not related to the number of NGO projects [6]. Unfortunately, the Bolivian data available for this analysis could not be used to test the relationship between NGO activity and poverty or population need at the community or individual level. When possible, future research should consider relative as well as absolute levels of poverty.

NGO activity was also found to be higher in those municipalities characterized by large indigenous populations. It may be the case that NGO activity is targeted towards populations considered vulnerable in a way that is easily identifiable namely, indigenous status, rather than towards poverty and poor health and social outcomes which are more difficult to assess and often require more advanced, technical, and resource intensive monitoring. It is interesting that we find an association between NGO activity and size of the indigenous population but not population need or poverty. Further research is needed to explore this finding in more detail.

Looking at the health sector, this study shows that NGO activity is related to health system coverage. However, the direction of the relationship is opposite to what would be expected if NGOs live up to the promise of being effective vehicles to fill the gaps in public sector coverage. We found higher NGO activity in municipalities with greater health system coverage. It may be the case that NGOs prefer to implement health related projects where the health system is functioning relatively well.

### Broader implications for global health and development

These results introduce two interesting questions relevant to broader discussions of global health and development research and practice. First, what does this uneven distribution of NGO activity in Bolivia suggest about the overall performance of the NGO sector? Secondly, considering that NGO activity may not be related to poverty or population need is this uneven distribution of NGO activity inequitable? It is important to note the distinction between inequality and inequity here: inequality is a descriptive term noting uneven distributions across social groups, classes, locations etc. while inequity involves a judgement as to whether such uneven distributions are also unjust [60]. It is difficult to answer these questions without appropriate benchmarks, guidelines and long-term surveillance to structure an evaluation of NGO performance and locate the possible inequities related to the NGO sector in general.

Although an evaluation of the NGO sector in Bolivia was not the goal of this paper (as data limitations inhibiting such an endeavour were recognized at the onset of this project), this point does raise an important issue. The current lack of benchmarks, standards, and guidelines for the NGO sector is a practical concern. Efforts are needed to create and implement national level guidelines that can be used as standards of appropriate operation and coverage for NGOs to follow and as evaluation benchmarks to assess the performance and equity of the NGO sector. An ‘NGO Code of Conduct’ that highlights priorities, encourages work in alignment with national development strategies, outlines standards of ethics, and satisfactory NGO coverage as well as possible benchmarks for evaluation and monitoring purposes is recommended. The concept of a code of conduct for the NGO sector has become more prevalent in health and development in the last decade. Several codes of conduct have been written, and, to varying degrees, adopted by organizations and nations. The ‘*NGO Code of Conduct for Health Systems Strengthening’* is a response to the proliferation of international NGO presence and is intended as a tool for service organizations – and eventually, funders and host governments [61]. Due to the difficulties in developing and implementing international strategies and guidelines, and recognizing the importance of country-specific context and needs, national level ‘NGO Codes of Conduct’ ought to be considered. Mozambique and Botswana are two nations that have created a code of conduct directed at the NGO sector (for example). The state, in collaboration with the NGO sector and other relevant stakeholders, is well advised to develop a national NGO Code of Conduct to guide NGO activity and the NGO sector. The public sector, the Ministry of Health for example, could play an important role in co-ordinating and monitoring adherence to a National NGO Code of Conduct. However, more work and research is needed to determine how a National NGO Code of Conduct could implemented in practice to ensure effectiveness and adherence to its principles. Additionally, a National NGO Code of Conduct could be a vehicle to improve co-ordination and harmonization of NGO efforts.

The general lack of surveillance of NGO related work and the NGO sector is concerning. The concept of surveillance, defined by the WHO as the ongoing, systematic collection, analysis, and interpretation of data essential to planning, implementation, and evaluation is worth considering here. The surveillance of NGO work should be improved at both the global and the national scale to better understand the impacts of the NGO sector. Large institutions and organizations such as the Global Fund, which have become the primary funding bodies for NGO work, should consider the importance of NGO surveillance and support and work towards creating accurate and representative databases that monitor their NGO-related activities at the international scale. At the national scale, NGO registries or databases similar to the one managed by the Bolivian government but with more detailed data pertaining to organizational and project characteristics should be encouraged and implemented. In countries such as Bolivia where an NGO registry does exists, efforts should be made to support and advance the collection, evaluation and dissemination of NGO related data. Additionally, registries would benefit from outlining what is defined as an NGO and as well as an NGO project, and making efforts to capture NGO and NGO project reach. Another option for surveillance and monitoring of the spatial distribution of NGO activity is to utilize mapping technology. Mapping technology is rapidly improving and could be used as a tool to monitor NGO work in a given nation or region.

As many authors have pointed out, the continued survival of the NGO sector may rest upon our ability to show that NGOs are performing and producing positive population level impacts [9,20,62]. This requires significant improvements in the surveillance and monitoring of the NGO sector.

## Conclusion

To begin to understand the municipal level contextual factors that shape the geographies of NGO activity across municipalities in Bolivia, we conducted regression analyses of count data. This study affirmed that NGO activity in Bolivia is unevenly distributed across space and found that NGOs do not appear to target their activities towards the poorest and neediest populations. It is however, difficult to evaluate overall performance of the NGO sector and determine whether NGO activity is equitably distributed without benchmarks and guidelines outlining national priorities and satisfactory NGO conduct and surveillance systems. The Bolivian National NGO Directory, although a valuable data source that has enabled this unique and original analysis of the Bolivian NGO sector, should aim to collect and make available more detailed data regarding the characteristics and reach of NGOs and their projects. Our findings suggest a need for efforts to ensure that resources are appropriately and effectively allocated to populations in need and standards and guidelines to frame evaluation and surveillance. They also suggest a need for improved communication and co-ordination between NGOs as well as funders of NGO work.

## Abbreviations

NGO: Nongovernmental organizations; LMICs: Low and middle income countries; UN: United Nations; GDP: Gross Domestic Product; UBN: Unsatisfied Basic Needs; IMR: Infant mortality rate; GIS: Geographic information system.

## Competing interests

The authors declare that they have no competing interests.

## Authors' contributions

LPG developed the study, carried out the data analyses and interpretation, and wrote the manuscript. LZ participated in the design of the study and provided statistical guidance and review. KC contributed to conceptual development, interpretation of results and writing and editing of the manuscript. All authors read and approved the final manuscript.
